# Methadone maintenance treatment is more effective than compulsory detoxification in addressing gut microbiota dysbiosis caused by heroin abuse

**DOI:** 10.3389/fmicb.2023.1283276

**Published:** 2023-10-20

**Authors:** Peng Yan, Haotian Ma, Wenrong Tian, Jincen Liu, Xinyue Yan, Lei Ma, Shuguang Wei, Jie Zhu, Yongsheng Zhu, Jianghua Lai

**Affiliations:** ^1^NHC Key Laboratory of Forensic Science, College of Forensic Science, Xi’an Jiaotong University, Xi’an, China; ^2^National Biosafety Evidence Foundation, Bio-evidence Sciences Academy, Western China Science and Technology Innovation Harbor, Xi'an Jiaotong University, Xi'an, China

**Keywords:** heroin use disorder, compulsory detoxification, methadone maintenance treatment, gut microbiota, depression, *Actinomyces*, *Turicibacter*, *Weissella*

## Abstract

**Introduction:**

Heroin use disorder (HUD) is commonly accompanied by gut dysbiosis, but the roles of gut microbiota in HUD treatment, such as compulsory detoxification and methadone maintenance treatment (MMT), remain poorly understood.

**Methods:**

In this study, we performed 16 s rDNA and whole metagenome sequencing to analyze the gut microbial profiles of HUD patients undergoing heroin addiction, heroin withdrawal (compulsory detoxification), and MMT.

**Results:**

Our findings revealed that, compared to healthy controls, microbial diversity was significantly decreased in HUD patients who were in a state of heroin addiction and withdrawal, but not in those receiving MMT. We observed significant alterations in 10 bacterial phyla and 20 bacterial families in HUD patients, while MMT partially restored these changes. Whole metagenome sequencing indicated gut microbiota functions were significantly disrupted in HUD patients experiencing heroin addiction and withdrawal, but MMT was found to almost reverse these dysfunctions. In addition, we identified 24 featured bacteria at the genus level that could be used to effectively distinguish between healthy individuals and those with heroin addiction, heroin withdrawal, or receiving MMT. Furthermore, we found the relative abundance of *Actinomyces*, *Turicibacter* and *Weissella* were positively associated with the Hamilton Depression Scale score in different states of HUD patients.

**Discussion:**

This study provides evidence from the gut microbiota perspective that MMT is a more effective approach than compulsory detoxification for HUD treatment.

## Introduction

Opioid use disorder represents a significant global public health challenge, resulting in profound physical, psychological, and societal ramifications. The 2023 World Drug Report highlights opioids as the most lethal category of drugs, responsible for approximately two-thirds of drug-related fatalities (UNODC, 2023). Heroin is the most widely abused drug among all opioids. The morbidity of heroin use disorder (HUD) rises dramatically in the past years (Cicero et al., 2017). Compared to other opioids, HUD is associated with a poorer prognosis (Karakula et al., 2016). Therefore, elucidating the pathogenesis and exploring effective treatment strategies of HUD are essential prerequisites for curbing the spread of heroin abuse.

The interaction between the gut microbiota and the central nervous system (CNS), known as the gut-brain axis, forms an integrated information exchange system that connects the brain and gut (Tache and Saavedra, 2022). This interaction allows the gut microbiota to influence the homeostasis and plasticity of the CNS by microbial metabolites and immune mediators, thereby further modulating brain function and behavior (Dinan and Cryan, 2017; Cryan et al., 2019). Recent studies have revealed the potential role of gut microbiota and their metabolites in modulating addiction-related behavior induced by opioids. In mice, the induction of morphine-induced conditioned place preference (CPP) was found to be accompanied by an increase in *Verrucomicrobia* and a decrease in *Bacteroides* (Zhang et al., 2021); the inhibition of short-chain fatty acid metabolites, achieved through non-absorbable antibiotics-mediated gut microbiota knockdown, resulted in a reduction of morphine-induced CPP formation (Hofford et al., 2021). Human studies have also demonstrated that opioid abuse is associated with significant perturbation of the gut microbiota. Reduced abundances of *Roseburia* and *Bilophila* perhaps contributed to the development of opioid use disorders through the induction of gut inflammation and dysregulation of bile acid metabolism (Gicquelais et al., 2020). Alterations in the gut microbiome induced by opioids are linked to the disruption of epithelial integrity, bacterial translocation, and the upregulation of pro-inflammatory cytokines (Akbarali and Dewey, 2017). Chronic opioid exposure can compromise the integrity of the blood–brain barrier, potentially facilitating the neurobiological effects of peripheral inflammatory factors originating from the gut (Hutchinson et al., 2011; Zhu et al., 2023). Despite all this knowledge, there are still many unresolved key issues in this domain.

The interplay between the intricate processes of the intestines and the CNS is a complex phenomenon. Due to discrepancies in study design, participant demographics, and dietary patterns, conflicting results have emerged in certain studies exploring the association between opioids abuse and gut microbiota (Xu et al., 2017; Gicquelais et al., 2020). In addition, compulsory detention-based forced detoxification and community-based methadone maintenance treatment (MMT) are the most common approaches for HUD treatment in China (Yang and Giummarra, 2021). However, few studies have explored the relationship between HUD treatment and gut microbiota (Cruz-Lebron et al., 2021). Moreover, heroin abuse characteristics, such as dosage, administration route, and negative emotions, significantly influence the onset, development, and treatment of HUD. Regrettably, research on the correlation between these characteristics and gut microbiota is currently lacking. Technical limitations also affect the research process; most studies use 16S rDNA sequencing to investigate the intestinal microbiome; however, this technology mainly focuses on the composition, evolutionary relationship, and diversity of the community, which is not deep enough (Yap et al., 2021). Whole metagenome sequencing can be used to conduct in-depth studies at the gene and functional levels. A combination of 16S rDNA sequencing and whole metagenome sequencing can provide information on the composition, diversity, and function of microbial communities (Vatanen et al., 2016).

In this study, we used 16S rDNA and whole metagenome sequencing to examine stool samples obtained from 34 healthy controls and 106 HUD patients experiencing heroin addiction, heroin withdrawal (compulsory detoxification), and MMT. Our aim was to investigate the characteristics of microbial diversity, composition and function, and determine the relationship between the heroin abuse characteristics and gut microbiota in different states of HUD patients.

## Materials and methods

### Subjects

We included 106 HUD patients experiencing heroin addiction (addiction group, recruited from the Anti-narcotics Department of Xi’an Public Security Bureau, *n* = 36), heroin withdrawal (compulsory detoxification) (withdrawal group, withdrawal duration >6 months, recruited from the Lantian Compulsory Isolated Detoxification Center, *n* = 40), and MMT (methadone group, MMT duration >6 months, recruited from the Xi’an Mental Health Center, *n* = 30). Males were selected because they comprise a much higher proportion of drug abusers than women in China, accounting for 88.3% of the total population (Sun et al., 2014). The diagnosis of opioid addiction was based on the International Classification of Diseases10th Edition (ICD-10) criteria, medical history, and urine test results. The basic information and heroin abuse characteristics (rout of heroin use, average dose, age of onset, heroin abuse duration, heroin withdrawal duration and MMT duration) were collected. HUD patients with a history of amphetamine, ketamine, barbiturate, benzodiazepine, marijuana, alcohol, or other drug addiction, as well as those with pre-existing mental illness before heroin abuse, were excluded from this study. Healthy controls (control group, *n* = 34) were recruited from the Health Examination Center of the First Affiliated Hospital of Xi’an Jiaotong University. Basic information were recorded. The inclusion criterion for controls was the absence of drug or alcohol dependence. Participants with a history of mental illness were excluded from the control group.

Participants were also excluded if they were taking other prescribed medications that could affect the CNS; had a history of seizures, hematological diseases, or severe lung, liver, or kidney impairment; had chronic infections, malignancies, autoimmune diseases, or hyperthyroidism; or were taking immunosuppressive agents. Participants with learning disabilities, head injuries, or other symptomatic psychoses were excluded from the present study. All participants were smokers but consumed <20 cigarettes per day, therefore did not meet nicotine addiction criteria according to the ICD-10. Participants were excluded if they consumed milk >250 mL/week, drank tea or coffee >5 days/week, were picky eaters, or vegetarians. All participants self-identified as third generation Han Chinese from Shaanxi Province. Moreover, the 24-items Hamilton Depression Scale (HAMD-24) was used to assess the presence and severity of depressive symptoms across all participants. Written informed consent was obtained from all the participants. The study protocol was approved by the Ethics Committee of the Medical College of Xi’an Jiaotong University (NO. 20201019).

### 16S rDNA sequencing

Fecal samples were collected between 7:00 to 10: 30 a.m. DNA was extracted from fecal matter using a QIAamp® Fast DNA Stool Mini kit (Qiagen, Hilden, Germany), following standard protocol. The lysis conditions used in this protocol were optimized to increase the ratio of human to non-human DNA. The V4 region of the fecal DNA samples was selected for PCR amplification. The PCR products were purified and subjected to PCR with sequencing indices [Nextera XT Index Primers 1 (N7xx) and 2 (S5xx)]. The index PCR products were purified, quantified (Agilent 2,100 Bioanalyzer, CA, United States), normalized, and pooled. Finally, the library was sequenced on a MiSeq platform (Illumina Inc., San Diego, CA, USA) using Genesky Biotechnologies (Shanghai, China). All raw sequences from 16 s rDNA sequencing were deposited in the NCBI Sequence Read Archive under project PRJNA809663, and the SRA numbers are listed in the Supplementary Table S1.

### Whole metagenome sequencing

After the 16S rDNA sequencing, to further explore the changes in gut microbiota function, 48 samples (12 from control group, 11 from addiction group, 13 from withdrawal group, and 12 from methadone group) were selected for whole metagenome shotgun sequencing (Vatanen et al., 2016). Nebulization was used to shear the qualified DNA samples into smaller fragments. Then, the overhangs resulting from fragmentation were converted into blunt ends using T4 DNA polymerase, Klenow fragments, and T4 polynucleotide kinase. Adapters were ligated to the ends of the DNA fragments after adding an adenine (A) base to the 3′ end of the blunt phosphorylated DNA fragments. Short fragments were then removed using Ampure beads. An Agilent 2,100 Bioanalyzer and ABI StepOnePlus Real-Time PCR System were used to qualify and quantify the sample libraries. Qualified libraries were sequenced using the Illumina HiSeq platform. All resulting raw sequences were deposited in the NCBI Sequence Read Archive under project PRJNA808806, and the SRA numbers are listed in the Supplementary Table S1.

### Bioinformatic and statistical analysis

**Figure 1:**
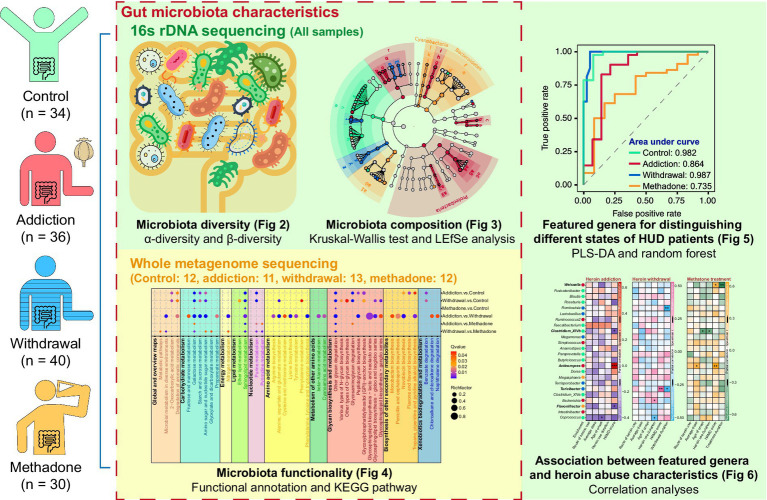
Schematic diagram of analysis process methodology. LEfSe: linear discriminant analysis (LDA) effect size; PLS-DA: partial least squares-discriminant analysis.

Based on previous microbiota-related research (Li et al., 2022; Blohs et al., 2023; Chen et al., 2023), this study first examined the differences in gut microbiota characteristics among different groups, including microbial diversity, composition, and functionality. Subsequently, a machine learning approach was employed to identify featured genera for distinguishing different states of HUD patients. Finally, correlation analysis was performed to confirm the correlation between these featured genera and the heroin abuse characteristics. The analysis process of this study is shown in Figure 1.

16S rDNA sequencing raw data were filtered to eliminate adapter pollution and low quality data to obtain clean reads, and then paired-end reads with overlaps were merged into tags. The tags were clustered into operational taxonomic units (OTUs) with 97% sequence similarity. OTU representative sequences were taxonomically classified using Ribosomal Database Project (RDP) Classifier trained on the database Greengene, using 0.6 confidence values as cut-off (DeSantis et al., 2006; Cole et al., 2009). Alpha diversity was analyzed based on OTU by using R package vegan, and the violin plots of these results were created by using GraphPad Prism 9 software. Principal coordinate analysis (PCoA) was performed using the R package vegan. Differential relative abundances of taxa were detected using Wilcoxon test (between healthy controls and all HUD patients) and Kruskal-Wallis test (between control, addiction, withdrawal and methadone groups), and the *p* values obtained were adjusted by the false discovery rate (FDR) method. To obtain results of pairwise comparison between groups, the Dunn post-hoc test was conducted on the taxa with FDR < 0.05 from the Kruskal-Wallis test. Linear discriminant analysis (LDA) effect size (LEfSe) was used to locate distinguishing taxa in different groups. Partial least squares-discriminant analysis (PLS-DA) was performed using the R package ropls. Random forest machine learning algorithm was conducted using the R package randomForest. Five 10-fold cross-validation trials were conducted to determine the optimal microbial biomarkers, with the cut-off point selected based on the mean of the minimum cross-validation error. All 140 samples were included in the random forest classifier, and each sample was tested only once. The samples within each group were randomly divided into a training set (60%) and a testing set (40%), while the remaining parameters were set to their default values. Receiver operating characteristic (ROC) curve was constructed, and the area under the curve (AUC) was calculated using the R package pROC. Co-occurrence networks analysis was performed using the R package ggClusterNet.

The qualified whole metagenome sequencing data produced using the Illumina platform were preprocessed. Raw data were assembled *de novo* with SOAPdenovo2 and Rabbit (Luo et al., 2012; You et al., 2013). MetaGeneMark was used to predict genes from assembled scaftigs (Zhu et al., 2010). The gene catalogs were blasted against public databases, including Clusters of Orthologous Groups (COG), Evolutionary Genealogy of Genes: Non-supervised Orthologous Groups (eggNOG), gene ontology (GO), and Kyoto Encyclopedia of Genes and Genomes (KEGG). Functional annotation and enrichment analyses were performed for differentially abundant genes. The potential biological pathways of the genes were illustrated using KEGG pathway enrichment.

Other statistical analyses were performed using GraphPad Prism (9.1) or R software (4.1.2). The parametric test (one-way ANOVA with Bonferroni’s post-hoc test) was applied when normality and homogeneity of variance assumptions were satisfied. The Kruskal-Wallis test with Dunn’s post-hoc test and chi-square (χ^2^) test were used for nonparametric tests. The Spearman’s bivariate correlation and partial correlation analyses were performed to detect the possible correlation. Statistical significance was set at *p* < 0.05. The codes related to visualization using R software were deposited in GitHub (https://github.com/haha11231/Gut-microbiota-and-HUD).

## Results

### Gut microbiota diversity in different states of HUD patients

**Figure 2:**
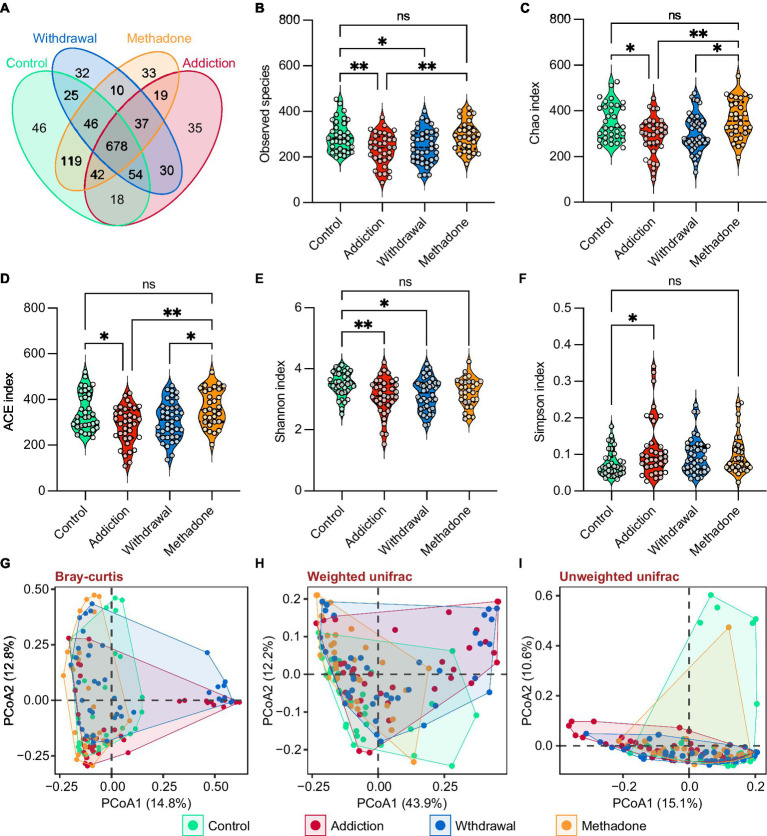
Gut microbiota diversity in different states of HUD patients. **(A)** Venn diagram showing the number of shared and unique OTUs among different groups. **(B-F)** Violin plots showing the α-diversity of gut microbiota among different groups, including observed species **(B)**, Chao **(C)**, ACE **(D)**, Shannon **(E)**, and Simpson **(F)** indexes; one-way ANOVA followed by the Bonferroni *post hoc* test, * *p* < 0.05, ** *p* < 0.01. **(G-I)** Principal coordinate analysis showing the β-diversity among different groups using bray-curtis **(G)**, weighted UniFrac **(H)**, and unweighted UniFrac **(I)** distance indexes; PERMANOVA test.

The general demographic characteristics of the recruited subjects are shown in Table 1. There were no significant differences among control, addiction, withdrawal and methadone groups in these characteristics. The Species Accumulation analysis showed that the majority of the microbiota could be detected in our sample pool (Supplementary Figure S1A). A total of 5,850,770 reads were obtained from all 140 fecal samples, with an average of 41,791 ± 601 clean reads per sample. High-quality paired-end reads were combined with tags based on overlapping. 5,838,650 tags were obtained, with 41,704 ± 573 tags per sample on average, and the average length was 282 ± 16 bp. The 140 samples produced 1,224 OTUs at a 97% similarity level (Supplementary Table S1). Shared and unique OTUs among the four groups are shown in Figure 2A and Supplementary Figure S1B.

**Table 1:** Demographic characteristics of the recruited subjects.

	Healthy controls (*n* = 34)	Heroin addiction (*n* = 36)	Heroin withdrawal (*n* = 40)	MMT (*n* = 30)	Analysis	Test details	*p* value
**Age (year, mean ± SD)**	39.68 ± 9.96	39.19 ± 12.28	40.70 ± 11.47	44.93 ± 10.36	Kruskal-Wallis test	Stat = 5.430	0.1429
**Education level (years)**					χ^2^ test	χ^2^ = 2.912, df = 3	0.4054
**≥ 9**	25	20	23	18			
**< 9**	9	16	17	12			
**Income (RMB)**					χ^2^ test	χ^2^ = 10.26, df = 6	0.1141
**≤2000**	5	13	16	14			
**2000–5,000**	23	20	20	15			
**≥5,000**	6	3	4	1			
**Martital status**					χ^2^ test	χ^2^ = 7.457, df = 6	0.2806
**Unmarried**	5	3	9	6			
**Married/cohabitating**	22	22	20	21			
**Divorced/separated**	7	11	11	3			
**Employment**					χ^2^ test	χ^2^ = 0.334, df = 3	0.9536
**Yes**	22	21	24	18			
**No**	12	15	16	11			
**Spicy foods**					χ^2^ test	χ^2^ = 1.183, df = 3	0.757
**Yes**	30	32	34	28			
**No**	4	4	6	2			
**Drink tea**					χ^2^ test	χ^2^ = 2.082, df = 3	0.5555
**Yes**	30	34	36	29			
**No**	4	2	4	1			

MMT, methadone maintenance treatment.

For α-diversity, the rarefaction curve based on the observed species (OBS), Chao, ACE, Shannon, and Simpson values indicated that the produced data were sufficient to cover all species in the community (Supplementary Figure S1C). The complexity of the sample was proportional to the OBS, Chao, ACE, and Shannon values but negatively correlated with the Simpson value. The OBS, Chao, ACE, and Shannon values were significantly lower in the addiction group than in the control group, the OBS and Shannon values were significantly lower in the withdrawal group than in the control group, and the Simpson value only increased in the addiction group, suggesting that bacterial diversity decreased after heroin addiction and withdrawal (Figures 2B–F). Interestingly, bacterial diversity was recovered after methadone treatment, as reflected by the lack of statistical differences in the OBS, Chao, ACE, Shannon, and Simpson values between the control and methadone groups (Figures 2B–F). In addition, the β-diversity was significantly different between the four groups, and PCoA showed separated clustering patterns at the OTU level, suggesting the greatest amount of variability in the entire dataset could be explained by the groups (PERMANOVA: bray-curtis, *R^2^* = 0.079, *p* = 0.001; unweighted unifrac, *R^2^* = 0.086, *p* = 0.001; weighted unifrac, *R^2^* = 0.105, *p* = 0.001) (Figures 2G–I and Supplementary Table S2).

### Altered gut microbiota composition in different states of HUD patients

**Figure 3:**
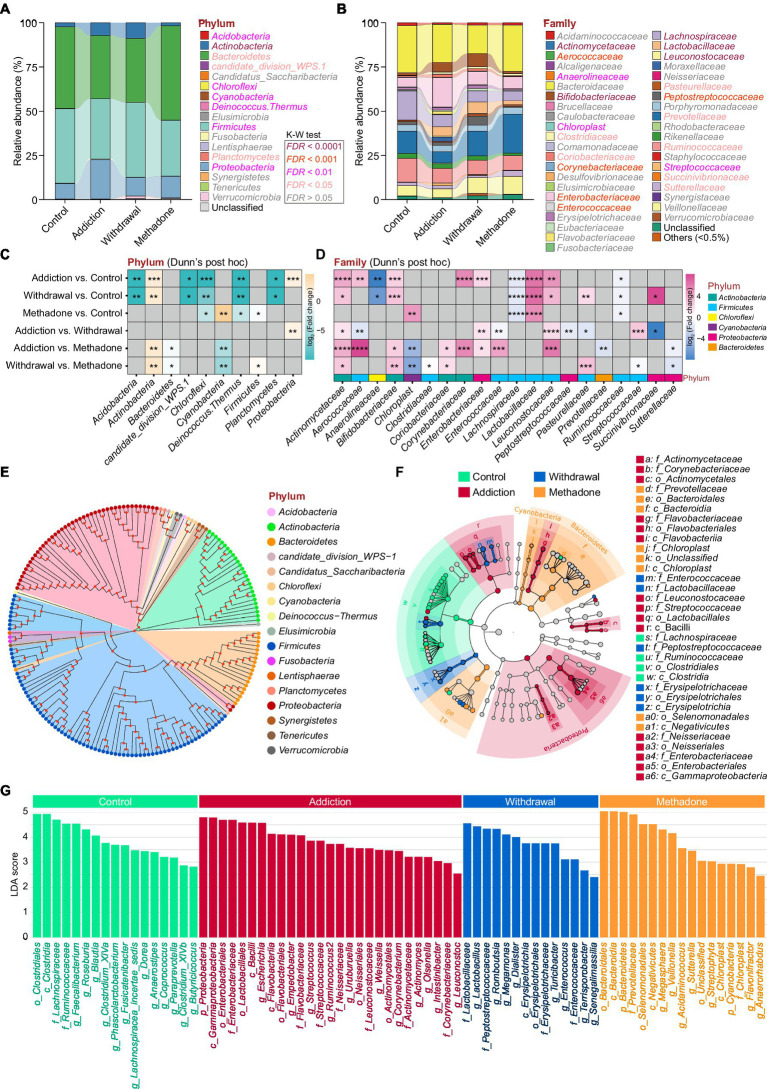
Altered gut microbiota compositions of different states of HUD patients. **(A-B)** Relative abundance of microbiota at the phylum **(A)** and family **(B)** levels among different groups; the differentially abundant taxa determined by the Kruskal-Wallis test (K-W test) are highlighted with colors. **(C,D)** Heatmap showing the between-group comparisons of differentially abundant phyla **(C)** and families **(D)**, identified using Dunn’s *post hoc* test, and the magnitude of change is quantified and colored by log_2_(fold change); * *p* < 0.05, ** *p* < 0.01, *** *p* < 0.001 and **** *p* < 0.0001. **(E)** Phylogenetic tree at the genus level, phyla that are the same are shown as the same color. **(F)** Linear discriminant analysis effect size (LEfSe) cladogram representing differentially abundant taxa among different groups. **(G)** A total of 75 differentially abundant taxonomic clades with LDA score > 2.0 and *p* < 0.05 were found among different groups.

Heroin profoundly influences gut microbiota composition. We observed differences in the abundance of 10 bacterial phyla, mainly including *Actinobacteria*, *Bacteroidetes*, *Firmicutes,* and *Proteobacteria* (Figure 3A), and 20 bacterial families, mainly including *Enterobacteriaceae*, *Lachnospiraceae*, *Prevotellaceae*, and *Ruminococcaceae* (Figure 3B). In the pairwise comparisons across the four different groups, larger differences in microbial composition were observed between the addiction and control groups, and between the withdrawal and control groups, both at the phylum and family levels. Interestingly, changes in the microbial composition between patients experiencing heroin addiction and withdrawal were more significant at the family level than at the phylum level. The difference in microbiota composition between the methadone and control groups was minimal (Figures 3C,D and Supplementary Table S3). In addition, significant differences were observed in the relative abundance of 11 classes, 12 orders, 51 genera, and 92 species across the four groups (Supplementary Figure S2 and Supplementary Table S3). The differences in gut microbiota composition between the healthy controls and all HUD patients were also analyzed and presented in Supplementary Table S3. Figure 3E illustrates the evolutionary relationship of the identified bacteria at the genus level, and most bacteria belonged to *Actinobacteria*, *Bacteroidetes*, *Firmicutes,* and *Proteobacteria*.

The relative abundances of all taxonomic groups were statistically compared at each taxonomic level among groups using the LEfSe algorithm on the Galaxy browser, and statistically abundant taxa are shown in Figures 3F and Supplementary Figure S3. LEfSe detected 75 enriched bacterial taxonomic clades across the four groups (Figure 3G). The control group was highly abundant in the order *Clostridiales*, notably in the families *Lachnospiraceae* and *Ruminococcacea*. The addiction group showed unique features, with an increase in the orders *Actinomycetales*, *Flavobacteriales* and *Neisseriales*, family *Streptococcaceae,* and *Leuconostocaceae*. We also found that the order *Erysipelotrichales* and families *Enterococcaceae*, *Lactobacillaceae,* and *Peptostreptococcaceae* were enriched in the withdrawal group. Meanwhile, the orders *Bacteroidales* and *Selenomonadales*, and family *Chloroplast* were enriched in the methadone group. These results suggest that the bacterial communities established in healthy individuals were replaced by subdominant groups in different states of HUD patients.

### Whole metagenome analysis of gut function characterization in different states of HUD patients

**Figure 4:**
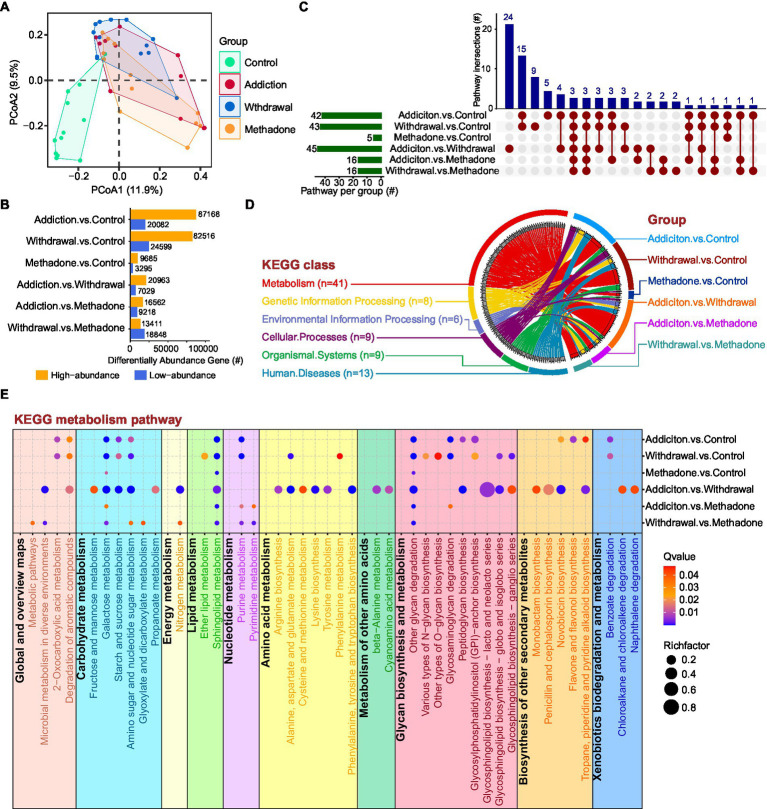
Whole metagenome analysis of gut function characterization of HUD patients in different states. **(A)** Principal coordinate analysis among different groups based on gene abundance. **(B)** Statistical analysis of genes with different abundances between different comparisons. The Y-axis represents pairwise comparisons and the X-axis represents the number of screened genes; the blue bar denotes low-abundance genes and the orange bar indicates genes with high-abundance. **(C)** UpSet plot showing the shared and unique KEGG enriched pathways between different comparisons. **(D)** Circos plot showing an overview of the differential pathway KEGG enrichment analysis among different comparisons. On the left side, the colors of the outer circle represent different KEGG classifications; the gray blocks on the inner circle represent different KEGG pathways, and the thickness of each gray block represents the enrichment number of the comparison groups, ranging from 1 to 6. On the right side, the colors of the outer circle represent different comparisons; the colors of the inner circle represent different KEGG classifications, and the thickness of each block represents the enrichment number of the KEGG pathways. Each curved line in the centre represents a KEGG pathway, connecting different KEGG classifications and comparison groups. **(E)** Bubble diagram showing the KEGG enriched pathways within the metabolism category among different comparisons. The RichFactor refers to the ratio of the number of differential genes located in the pathway to the total number of genes annotated in the pathway; the larger the RichFactor, the greater the degree of enrichment.

To further explore the functional differences in the gut microbiota among different states of HUD patients, 48 samples were selected for shotgun whole metagenome sequencing (Vatanen et al., 2016). After *de novo* metagenomic assembly, a non-redundant catalog of 2,373,114 genes was constructed. The length distributions of these genes are shown in Supplementary Figure S4A. PCoA analysis showed significant dissimilarity between groups at the gene level (PERMANOVA: Bray-Curtis, *R^2^* = 0.150, *p* = 0.001), but no significant difference was observed between heroin-addicted and methadone-treated individuals (*p* = 0.205) (Figure 4A and Supplementary Table S2). Figure 4B shows the total list of differentially abundant gene numbers between groups, and the addiction vs. control and withdrawal vs. control groups had more differential genes than the other comparisons. To obtain more information, the genes were blasted against COG, eggNOG, GO, and KEGG databases, and a summary of the gene annotations is shown in Supplementary Figures S4B–E.

Next, KEGG pathway enrichment analysis was performed to assess the systems view of microbial function affected by heroin addiction, heroin withdrawal and methadone treatment. In the pairwise comparisons across the four different groups, differentially abundant genes in the addiction vs. control, withdrawal vs. control, and addiction vs. withdrawal groups were enriched in a greater number of pathways, while methadone vs. control was enriched in the fewest pathways (only 5) (Figure 4C). The upSet plot shows the shared differentially enriched pathways between each comparison, addiction vs. withdrawal had the highest number of unique differentially enriched pathways, and three pathways were enriched in all comparisons (Figure 4C). The Circos plot shows the abundance relationship between the differentially enriched pathways and KEGG classifications, with the metabolism category being the most enriched for each comparison (Figure 4D). Within this category, multiple pathways were enriched, including carbohydrate, energy, lipid, nucleotide, amino acid metabolism, glycan biosynthesis and xenobiotic biodegradation (Figure 4E). Addiction vs. control and withdrawal vs. control had similar coverage in metabolic pathways, except for amino acid metabolism and biosynthesis of other secondary metabolites; addiction vs. withdrawal had the highest coverage in metabolic pathways (Figure 4E). Interestingly, the methadone group-related comparisons had lower coverage of metabolic pathways, especially methadone vs. control (Figure 4E). These results suggest that the microbial shifts induced by addiction, withdrawal, and methadone treatment were accompanied by altered expression of functional genes in the community.

### Featured microbiota for distinguishing different states of HUD patients

**Figure 5:**
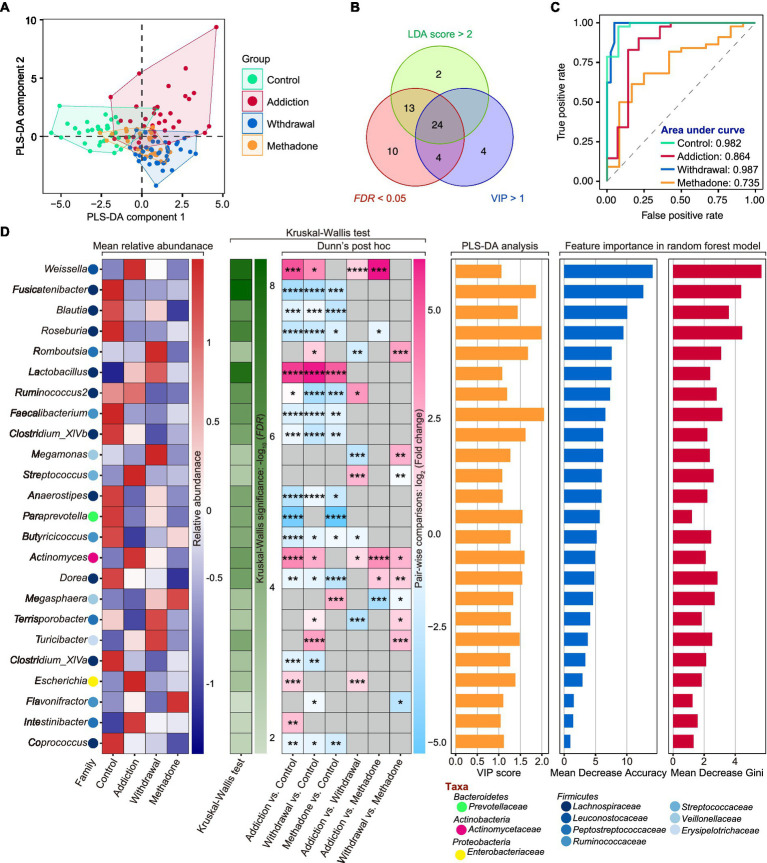
Featured microbiota for distinguishing different states of HUD patients. **(A)** Partial least squares-discriminant analysis (PLS-DA) score plot of gut microbiota at the genus level among different groups. **(B)** A total number of 24 featured bacteria genera were selected according to the Variable Importance for the Projection (VIP) score in PLS-DA (> 1), LDA score in LEfSe (> 2 and *p* < 0.05), and FDR value (< 0.05) in Kruskal-Wallis test. **(C)** The area under the curve (AUC) value of receiver operating characteristic (ROC) curve analysis reflects the effectiveness of the 24 featured bacteria genera in discriminating between different states of HUD patients using the random forest model. **(D)** The detailed information of these featured bacteria genera including mean relative abundance, results of differential analysis, VIP score and the score of mean decrease accuracy and mean decrease Gini; * *p* < 0.05, ** *p* < 0.01, *** *p* < 0.001 and **** *p* < 0.0001.

We then wanted to identify signature bacteria that could discriminate between healthy, heroin-addicted, heroin-withdrawn, and methadone-treated individuals at the genus level based on the results of 16S rDNA sequencing. To this end, we first implemented PLS-DA and calculated Variable Importance for the Projection (VIP) scores based on the relative abundance of genera. PLS-DA is a supervised classification model with superior resolution capability and efficacy, and is often used for variation identification. The VIP score is considered the weight for estimating the contribution of the model, and a VIP > 1 is the criterion for identifying significant variations in the model (Bai et al., 2022). The PLS-DA plot exhibited a distinct group clustering pattern (*R*^2^X = 0.141, *R*^2^Y = 0.459, Q^2^ = 0.260), and 33 genera with VIP > 1 were found (Figure 5A and Supplementary Table S4). Next, a total of 24 featured bacteria were selected based on the dominant bacteria (LEfSe: LDA score > 2 and *p* < 0.05) and differential analysis (Kruskal-Wallis test: FDR values <0.05) (Figure 5B). Finally, these featured bacteria were used to build a multi-classification model using the random forest machine learning algorithm.

The ROC curve and AUC were used to evaluate the model performance. After five 10-fold cross-validation trials, all featured bacteria were retained with optimal performance, based on which a multi-classification model was established (Supplementary Figure S5). The random forest classifier could effectively identify healthy controls with an AUC of 0.982 and an accuracy of 0.945; heroin addicts with an AUC of 0.864 and an accuracy of 0.836; heroin withdrawn individuals with an AUC of 0.987 and an accuracy of 0.857; and methadone-treated individuals with an AUC of 0.735 and an accuracy of 0.732 (Figure 5C). The mean relative abundance, differential analysis results (Kruskal-Wallis test), VIP scores in PLS-DA, and importance in random forest classifier of the 24 featured bacteria are shown in Figure 5D. The main taxa were the phylum *Firmicutes*, and the top5 important genera were *Weissella*, *Fusicatenibacter*, *Blautia*, *Roseburia,* and *Romboutsia* (the larger the Mean Decrease Gini and Mean Decrease Accuracy, the more important they are). These results indicate that the random forest classifier containing the 24 featured bacteria was able to discriminate between healthy, heroin-addicted, heroin-withdrawn, and methadone-treated individuals with good performance.

### Featured genera associated with heroin abuse characteristics in different states of HUD patients

**Figure 6:**
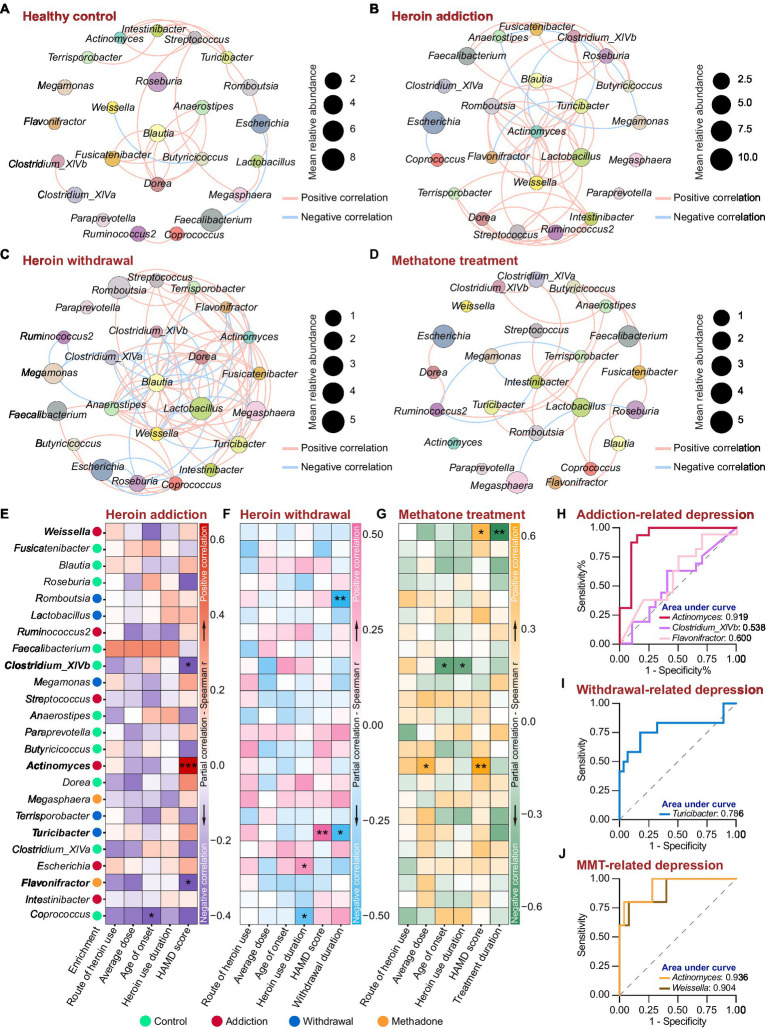
Associations between the featured genera and heroin abuse characteristics in different states of HUD patients. **(A-D)** The co-occurrence networks based on Spearman’s correlations between the 24 featured genera in the control **(A)**, addiction **(B)**, withdrawal **(C)** and methadone **(D)** groups. The size of the nodes represents the mean relative abundance. Edges represent positive (red) and negative (blue) correlations. Only significant correlations (r > |0.4| and *p* < 0.05) are shown. **(E-G)** Partial correlation between the relative abundance of the featured genera and heroin abuse characteristics by Spearman’s partial correlation analysis in addiction **(E)**, withdrawal **(F)** and methadone **(G)** groups. The species enrichment in different groups based on the LEfSe analysis are shown on the left. Detailed controlling variables are shown in Supplementary Table S7. Red, pink and orange indicate positive correlation, and darkblue, skyblue and green mean negative correlation. * *p* < 0.05, ** *p* < 0.01 and *** *p* < 0.001. **(H-J)** ROC curve analysis using the relative abundance of certain genera within the 24 featured bacteria to predict HUD-related depression in addiction **(H)**, withdrawal **(I)** and methadone **(J)** groups.

We next analyzed the association between the 24 featured genera and the heroin abuse characteristics. The co-occurrence networks were generated based on Spearman’s bivariate correlations among the relative abundances of the featured genera. There was an obvious difference in the network structure among groups, and heroin addiction and withdrawal strongly increased the complexity of the networks (Figures 6A–D and Supplementary Table S5). In the control and withdrawal groups, *Blautia* was positioned at the centre of the co-occurrence network (Figures 6A,C); while in the addiction and methadone groups, *Actinomyces* and *Intestinibacter* occupied the central position, respectively (Figures 6B,D). The heroin abuse characteristics of HUD patients are displayed in Table 2, and the HAMD score in the methadone group was significantly lower than that in the addiction and withdrawal groups. There was a significant correlation between average usage dose and route of heroin use. Both age of onset and heroin use duration were positively associated with age at the time of recruitment (Supplementary Figure S6A and Supplementary Table S6). In addition, the HAMD score was negatively associated with the MMT duration in the methadone group (Supplementary Figure S6B and Supplementary Table S6).

**Table 2:** Heroin abuse characteristics of the HUD patients.

	Heroin addiction	Heroin withdrawal	MMT	Analysis	Test details	*p* value
**Route of heroin use**				χ^2^ test	χ^2^ = 0.5555, df = 4	0.9697
**Snorting**	19	21	17			
**Intravenous**	13	16	10			
**Mixed**	4	3	3			
**Average dose (g/day, mean ± SD)**	0.7861 ± 0.6076	0.7425 ± 0.5697	0.8467 ± 0.5929	Kruskal-Wallis test	Stat = 0.5821	0.7475
**Age of onset (year, mean ± SD)**	27.61 ± 7.392	28.53 ± 6.782	30.87 ± 7.099	One-way ANOVE	*F*_(2,103)_ = 0.1974	0.8212
**Heroin use duration (year, mean ± SD)**	11.58 ± 8.087	12.18 ± 8.75	14.07 ± 7.991	Kruskal-Wallis test	Stat = 1.952	0.3769
**HAMD score (mean ± SD)**	20.61 ± 11.27	16.55 ± 8.391	10.73 ± 6.47^†††, ‡^	Kruskal-Wallis test	Stat = 16.22	0.0003
**Withdrawal duration (month, mean ± SD)**	/	9.2 ± 2.053	/	/	/	/
**MMT duration (month, mean ± SD)**	/	/	9.2 ± 1.919	/	/	/

MMT, methadone maintenance treatment.

^†††^ *p* < 0.001 compared with heroin addiction group, ^‡^ *p* < 0.05 compared with heroin withdrawal group, Dunn’s post hoc test.

Spearman’s partial correlation analysis (controlling for demographic and heroin abuse characteristics, detailed controlling variables are shown in Supplementary Table S7) was used to examine the associations between the relative abundance of featured genera and the heroin abuse characteristics in different states of HUD patients. In the addiction group, there was a mutual correlation among *Actinomyces*, *Clostridium*_XlVb and *Flavonifractor* (Figure 6A); *Actinomyces* was more abundance in addiction group and was positively associated with the HAMD score, while *Clostridium*_XlVb and *Flavonifractor* were negatively associated with the HAMD score (Figure 6E); *Coprococcus* was negatively associated with the age of onset (Figure 6E). In the withdrawal group, *Turicibacter* was abundant in withdrawal group, and a higher relative abundance of *Turicibacter* was correlated with a higher HAMD score (Figure 6F); there was a significant positive correlation between *Turicibacter* and *Romboutsia* (Figure 6B), and both of which were negatively associated with the heroin withdrawal duration (Figure 6F); *Escherichia* and *Coprococcus* were positively and negatively associated with heroin use duration, respectively (Figure 6F). In the methadone group (Figure 6G), *Actinomyces* was positively associated with both average usage dose and HAMD score; *Clostridium_XlVb* was negatively associated with both heroin use duration and age of onset; *Weissella* was positively associated with HAMD score and negatively associated with methadone treatment duration.

Using a HAMD score of 21 as the threshold, we performed ROC curve analysis to assess the effectiveness of the relative abundance of *Actinomyces*, *Clostridium*_XlVb, *Flavonifractor*, *Turicibacter* and *Weissella* in predicting depression in different states of HUD patients. Compared to *Clostridium*_XlVb and *Flavonifractor*, the relative abundance of *Actinomyces* showed better predictive efficacy for depression in the addiction group (*Actinomyces*: AUC = 0.9188, *p* < 0.0001; *Clostridium*_XlVb: AUC = 0.538, *p* = 0.7024; *Flavonifractor*: AUC = 0.600, *p* = 0.3083) (Figure 6H and Supplementary Table S8). The relative abundance of *Turicibacter* demonstrated good diagnostic power for depression in the withdrawal group (AUC = 0.786, *p* < 0.01) (Figure 6I and Supplementary Table S8). The relative abundance of *Actinomyces* and *Weissella* had an excellent predictive efficacy for depression in the methadone group (*Actinomyces*: AUC = 0.936, *p* < 0.01; *Weissella*: AUC = 0.904, *p* < 0.01) (Figure 6J and Supplementary Table S8). These results indicate that heroin addiction and withdrawal had a great influence on the co-occurrence networks of the featured genera; there was a significant correlation between the relative abundances of the featured genera and heroin abuse characteristics, especially for HAMD score; and *Actinomyces*, *Turicibacter* and *Weissella* could be used as biomarkers to predict depressive symptoms in different states of HUD patients.

## Discussion

In this study, we investigated the relationship between the gut microbiota and HUD using 16S rDNA and whole metagenome sequencing. We found that heroin addiction and withdrawal significantly altered the gut microbial diversity, composition, and function, whereas methadone treatment could somewhat reverse these negative effects. In addition, we identified 24 featured bacteria that could be used to distinguish between healthy, heroin-addicted, heroin-withdrawal, and methadone-treated individuals with high reliability. Meanwhile, to our best knowledge, this is the first study to investigate the associations between gut microbiota and HUD-related depression, and found *Actinomyces*, *Turicibacter* and *Weissella* were significantly associated with HAMD score in different states of HUD patients.

Reduced microbial diversity is a typical characteristic for microbial dysbiosis, and has been observed in various neuropsychiatric diseases (Cryan et al., 2019; Socala et al., 2021; Sorboni et al., 2022). Previous preclinical and clinical studies indicated opioid agonist use, such as morphine and heroin, results in decreased alpha diversity of microbiota (Wang et al., 2018; Gicquelais et al., 2020). Consistent with this, our results confirmed that heroin significantly impacted the diversity of the human gut microbiome. A previous study reported increased alpha diversity in 45 Chinese men with substance use disorder (58% used heroin) (Xu et al., 2017). In our study, only patients exhibiting heroin abuse were recruited, which may explain the differences in the findings. In addition, we found that heroin withdrawal individuals who had been through drug abstinence for more than six months still had a lower alpha diversity, but the HUD patients receiving MMT showed reversed microbial diversity. Opioid use causes global alterations in gut microbiota, but little is known about gut microbiota alternations undergoing opioid withdrawal and MMT (Acharya et al., 2017; Barengolts et al., 2018; Gicquelais et al., 2020; Li et al., 2020; Cruz-Lebron et al., 2021). Our data revealed that the addiction group vs. control group and withdrawal group vs. control group had a similar differential abundance pattern, especially at the phylum level, including a preferential increase in *Actinobacteria* and decrease in *Acidobacteria*, *candidate_division_WPS.1*, *Chloroflexi*, *Deinococcus.Thermus,* and *Planctomycetes*; however, the bacterial compositions between the addiction and withdrawal groups remained significantly different at the family level; MMT greatly improved bacterial dysbiosis in patients with HUD. These results suggest that MMT, rather than compulsory detoxification, partially recovers heroin-induced changes in the microbiota composition. Microbial diversity and compositions affect brain function and behavior (Yano et al., 2015; Kiraly et al., 2016), although recovering intestinal diversity after heroin administration is challenging, it is still key to successful HUD therapy.

Using whole metagenome sequencing analysis, we identified 86 differentially enriched pathways from six KEGG categories between the different states of HUD patients. Among these pathways, 41 belonged to metabolism, mainly involving carbohydrate, amino acid and glycan metabolism. Opioids not only disrupt the intestinal barrier by altering gut microbiota composition (Wang et al., 2018; Simpson et al., 2022), but also increase blood–brain barrier (BBB) permeability (Zhu et al., 2023). This provides an avenue for microbial metabolites from the gut to cross the damaged BBB and interact with the CNS, leading to energy metabolic disturbances (Solis et al., 2017), abnormal neurotransmitter synthesis (Chen et al., 2021), and neuroinflammation (Bostick et al., 2022), which may affect both the development and treatment of HUD. Although new methods and modalities for the treatment of HUD, such as transcranial magnetic stimulation and deep brain stimulation, have been reported in recent years, compulsory detoxification and MMT remain the most commonly used approaches for HUD treatment in China (Mahoney et al., 2020). We found that heroin addiction and withdrawal significantly affected gut microbiota function. In the pairwise comparisons, the largest difference was found between addiction and withdrawal. These results suggest that even after more than six months of heroin withdrawal, HUD patients still experience serious gut microbiota dysbiosis, which may be a heroin withdrawal-specific gut change. A similar trend has also been observed in the immune system. Re et al. showed that immune dysregulation in HUD patients persisted for more than 12 months after heroin withdrawal (Re et al., 2022). These findings help explain the high relapse rate associated with compulsory detoxification (O'Sullivan et al., 2019). Surprisingly, our research revealed that methadone treatment has been shown to almost reverse abnormal gut microbiota function caused by heroin abuse, further demonstrating that MMT remains a viable option for HUD treatment (Bell and Strang, 2020).

Gut microbiota has emerged as a valuable biomarker for differentiating individuals with schizophrenia from those with bipolar disorder, and distinguishing patients with major depressive disorder from healthy controls (Liu et al., 2021; Chen et al., 2023). However, there is currently a lack of research investigating the potential of gut microbiota in distinguishing the different states of HUD patients. Based on PLS-DA, differential abundance, and LDA analyses, we identified 24 featured bacteria at the genus level, and submitted a random forest classification model for distinguishing between healthy, heroin-addicted, heroin-withdrawn, and methadone-treated individuals using these genera. AUC is an acceptable parameter to evaluate the effectiveness of the model, and AUC values greater than 0.7, 0.8 and 0.9 indicate fair, very good and excellent classification efficiency, respectively (Cicchetti, 2001; Wu et al., 2021). Thus, our model performed best in identifying healthy and heroin-withdrawn individuals, followed by identifying heroin-addicted and methadone-treated individuals. Moreover, in the future, it is advisable to improve the efficacy for distinguishing different states of HUD patients by incorporating additional clinical features, particularly imaging data, and employing advanced computational methods (Hu et al., 2023).

Alterations in microbial composition are the key reason for gut dysbiosis, and specific characteristics of the microbiome can indicate either detrimental or beneficial effects on HUD patients (Simpson et al., 2022). Therefore, we hypothesize that the 24 featured genera may have a more significant impact on HUD compared to other bacterial genera. Our findings demonstrate significant associations between certain genera within the 24 featured bacteria and heroin abuse characteristics. Among all these characteristics, we are particularly interested in the depressive symptoms, as it is a commonly observed comorbid psychiatric disorder in HUD patients and is known to significantly influence the treatment outcome and prognosis of HUD (Volkow et al., 2019; Tolomeo et al., 2021). In line with previous studies, we found a significant prevalence of depression in the addiction and withdrawal groups, but MTT significantly reduced depressive symptoms (Volkow et al., 2019; Moazen-Zadeh et al., 2021; Tolomeo et al., 2021; Xin et al., 2022). These findings suggest that MMT may improve the depressive symptoms in HUD patients by ameliorating the gut microbiota dysbiosis caused by heroin abuse, thereby enhancing the prognosis of HUD. Our study revealed a positive correlation between the increased abundance of *Actinomyces* and higher HAMD scores in both heroin addicts and individuals undergoing MMT, indicating a potential association between *Actinomyces* and opiate agonist-related depression. Additionally, our findings suggest that *Turicibacter* may specifically impact heroin withdrawal-related depression. The elevated abundance of *Actinomyces* and *Turicibacter* in depression patients supports their potential contribution to the pathophysiology of depression (Kelly et al., 2016; Barandouzi et al., 2020). Thus, despite HUD and depression being distinct disorders, there may exist shared gut microbiota and related gut mechanisms that play a role in both diseases. Surprisingly, we observed a positive correlation between the relative abundance of *Weissella* and MMT-related depression, which gradually decreased as the increased duration of MMT. Recent study has highlighted the promising probiotic properties of *Weissella* (Teixeira et al., 2021), but some strains from this genus may proliferate in immunocompromised individuals (Fusco et al., 2015). Therefore, we speculate that *Weissella* may act as an opportunistic pathogen in HUD patients, and the decrease in the relative abundance of *Weissella* may indicate the restoration of the intestinal immune environment. Furthermore, the relative abundances of the above three genera demonstrated strong predictive ability for HUD-related depression, suggesting their potential as biomarkers for assessing the prognosis of HUD.

This study has three limitations that need to be addressed. First, we did not detect metabolic changes in the different states of patients with HUD. Second, we only included male patients due to the scarcity of female HUD patients. Third, the analyses in this study predominantly concluded at the genus level due to the limitations of 16S rDNA sequencing in accurately resolving taxa beyond this level (Johnson et al., 2019; Blohs et al., 2023). However, we still propose a strong correlation between HUD and gut dysbiosis. Future studies should comprehensively analyze the role of gut microbiota (phyla to species), metabolites, and immunological parameters in HUD while considering host factors such as sex, genetics, and internal and external environments. Functional validation is also necessary to clarify the neurobiological mechanisms by which the gut microbiota regulates the development and treatment of HUD. Furthermore, using deep learning techniques to develop drugs targeting gut microbiota is a promising strategy for heroin addiction treatment (Zhao et al., 2022, 2023).

In summary, our study characterized the distinct gut microbiota profiles in HUD patients undergoing heroin addiction, heroin withdrawal and MMT. We found that MMT, rather than heroin withdrawal significantly improves the alterations in gut microbiota diversity, composition, and function caused by heroin abuse. Additionally, we submitted a classification model including 24 feature genera that can accurately distinguish the different states of HUD patients. Furthermore, our findings indicate that *Actinomyces*, *Turicibacter* and *Weissella* have shown potential as biomarkers for predicting HUD-related depression symptoms. Our work emphasizes the superiority of MMT over compulsory detoxification in the HUD treatment, and suggests restoring gut dysbiosis to improve HUD-related depression may represent a novel and effective therapeutic strategy for HUD.

## Data availability statement

The datasets presented in this study can be found in online repositories. The names of the repository/repositories and accession number(s) can be found in the article/Supplementary material.

## Ethics statement

The studies involving humans were approved by Ethics Committee of the Medical College of Xi’an Jiaotong University. The studies were conducted in accordance with the local legislation and institutional requirements. The participants provided their written informed consent to participate in this study.

## Author contributions

PY: Conceptualization, Data curation, Formal Analysis, Funding acquisition, Methodology, Resources, Visualization, Writing – original draft, Writing – review & editing. HM: Data curation, Formal analysis, Investigation, Methodology, Validation, Visualization, Writing – original draft. WT: Data curation, Investigation, Writing – original draft. JLi: Data curation, Investigation, Validation, Writing – original draft. XY: Data curation, Investigation, Writing – original draft. LM: Data curation, Investigation, Writing – original draft, SW: Funding acquisition, Resources, Supervision, Validation, Writing – original draft. JZ: Conceptualization, Methodology, Project administration, Supervision, Validation, Writing – original draft. YZ: Conceptualization, Data curation, Formal Analysis, Investigation, Methodology, Project administration, Writing – original draft, Writing – review & editing. JLa: Conceptualization, Funding acquisition, Methodology, Project administration, Resources, Supervision, Validation, Writing – original draft, Writing – review & editing.
