# Supplementary material for: Methadone maintenance treatment is more effective than compulsory detoxification in addressing gut microbiota dysbiosis caused by heroin abuse
Source: Front Microbiol. 2023 Oct 20;14:1283276. doi: 10.3389/fmicb.2023.1283276 (PMC10635210; doi:10.3389/fmicb.2023.1283276)
Supplement: Supplementary file 2 [file Data_Sheet_1.PDF]

Supplementary Figures

Methadone maintenance treatment is more effective than compulsory detoxification in addressing gut microbiota dysbiosis caused by heroin abuse

Peng Yan, Haotian Ma, Wenrong Tian, Jincen Liu, Xinyue Yan, Lei Ma, Shuguang Wei, Jie Zhu, Yongsheng Zhu, Jianghua Lai

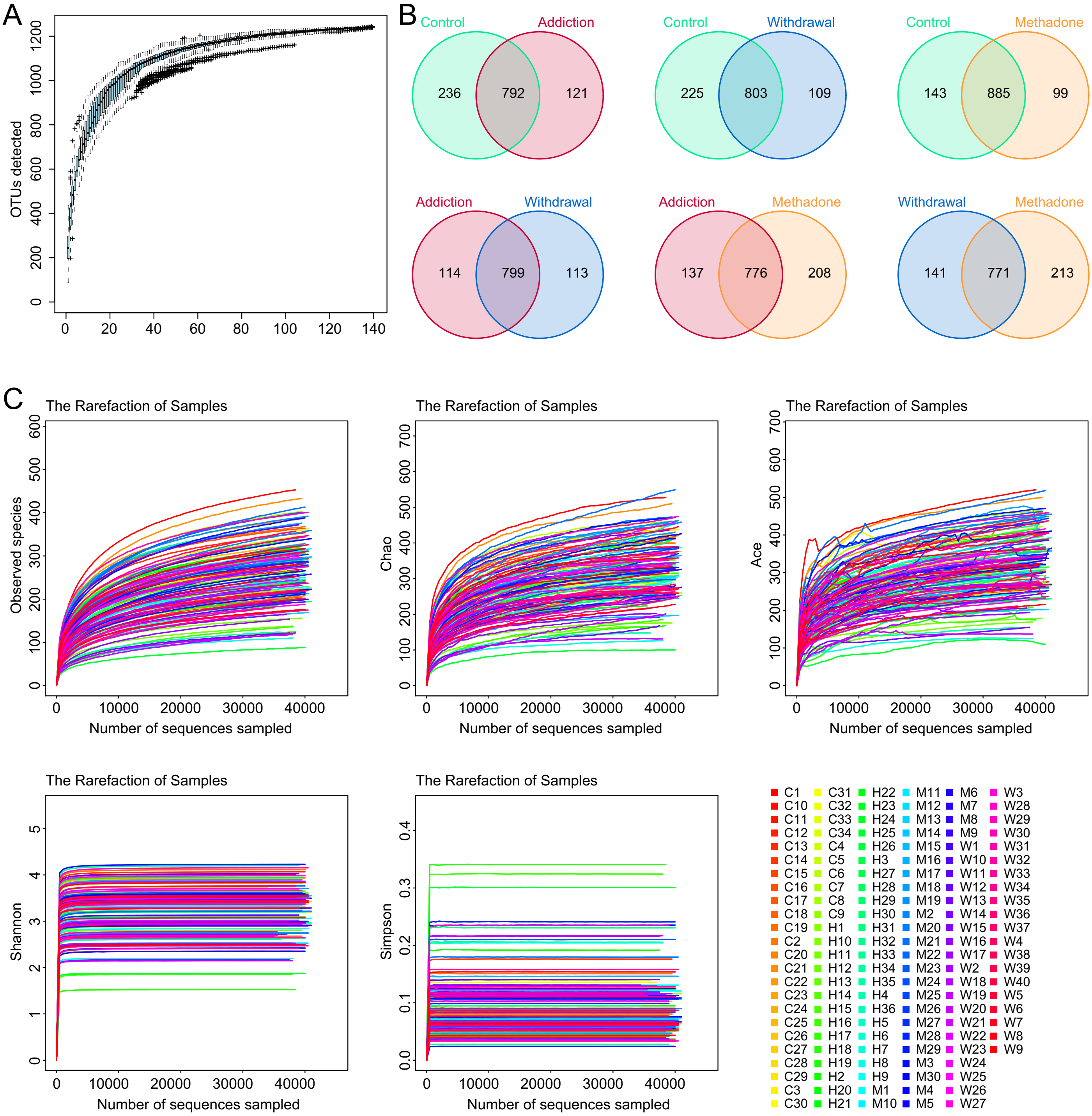

Figure S1. OTU cluster information of HUD patients in different states. (A) Species Accumulation (SA) analysis. SA plots show the increase in OTUs detected with the addition of each sample. Each bar represents 100 random draws (without replacement) of samples from the sample pool. The figure shows the curve obtained using all of the OTU data. (B) The number of microbial OTUs shared between-group comparisons. (C) Sample-based rarefaction analysis, including observed species, Chao, ACE, Shannon, and simpson indexes; C: healthy controls, H: heroin addiction, W: heroin withdrawal, M: methadone treatment.

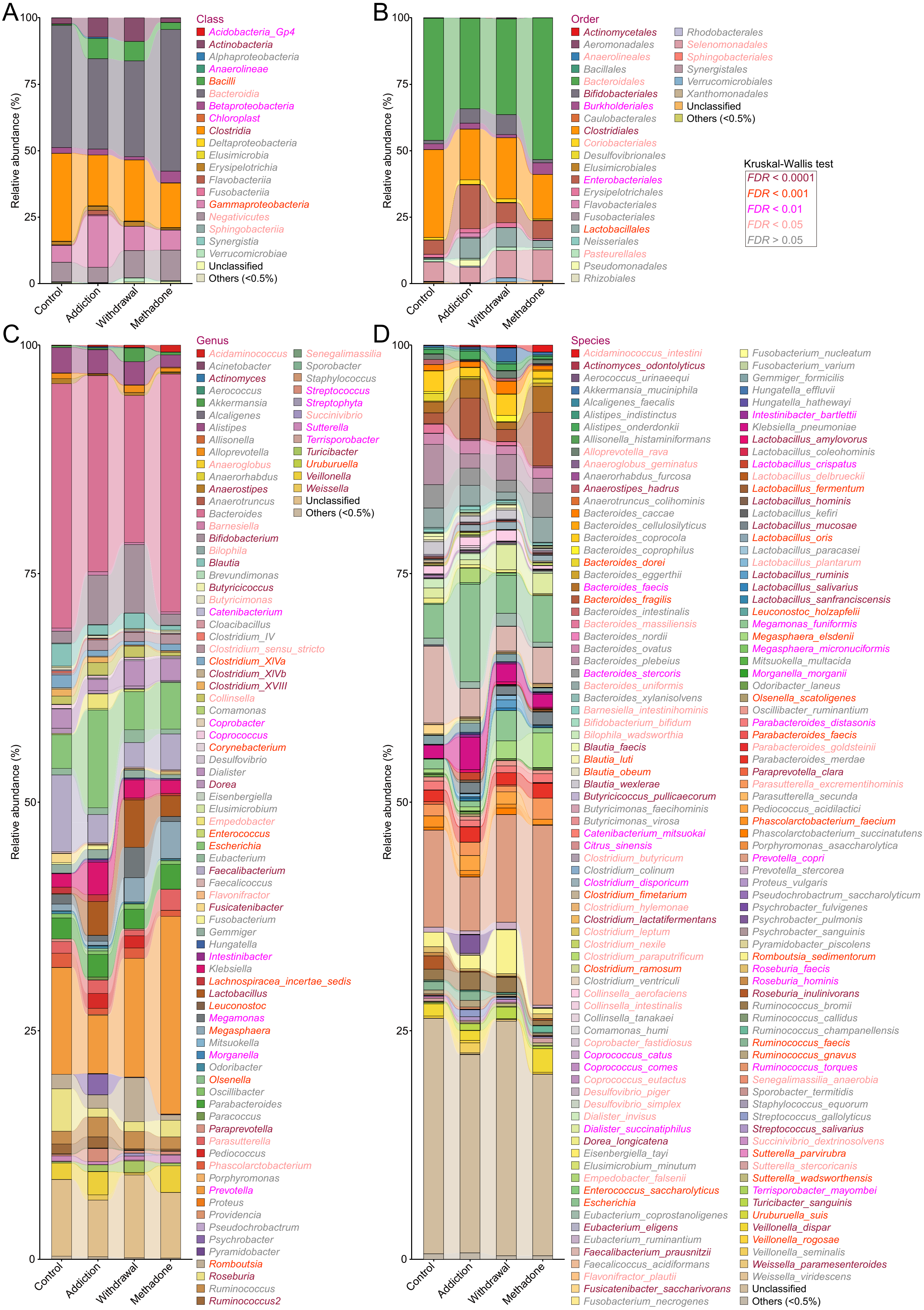

Figure S2. **Altered gut microbiota composition at the class, order, genus and phylum levels.** Relative

abundance of microbiota compositions at the class (A), order (B), genus (C) and phylum (D) levels among different groups; the differentially abundant taxa determined by the Kruskal-Wallis test are highlighted with different colors.

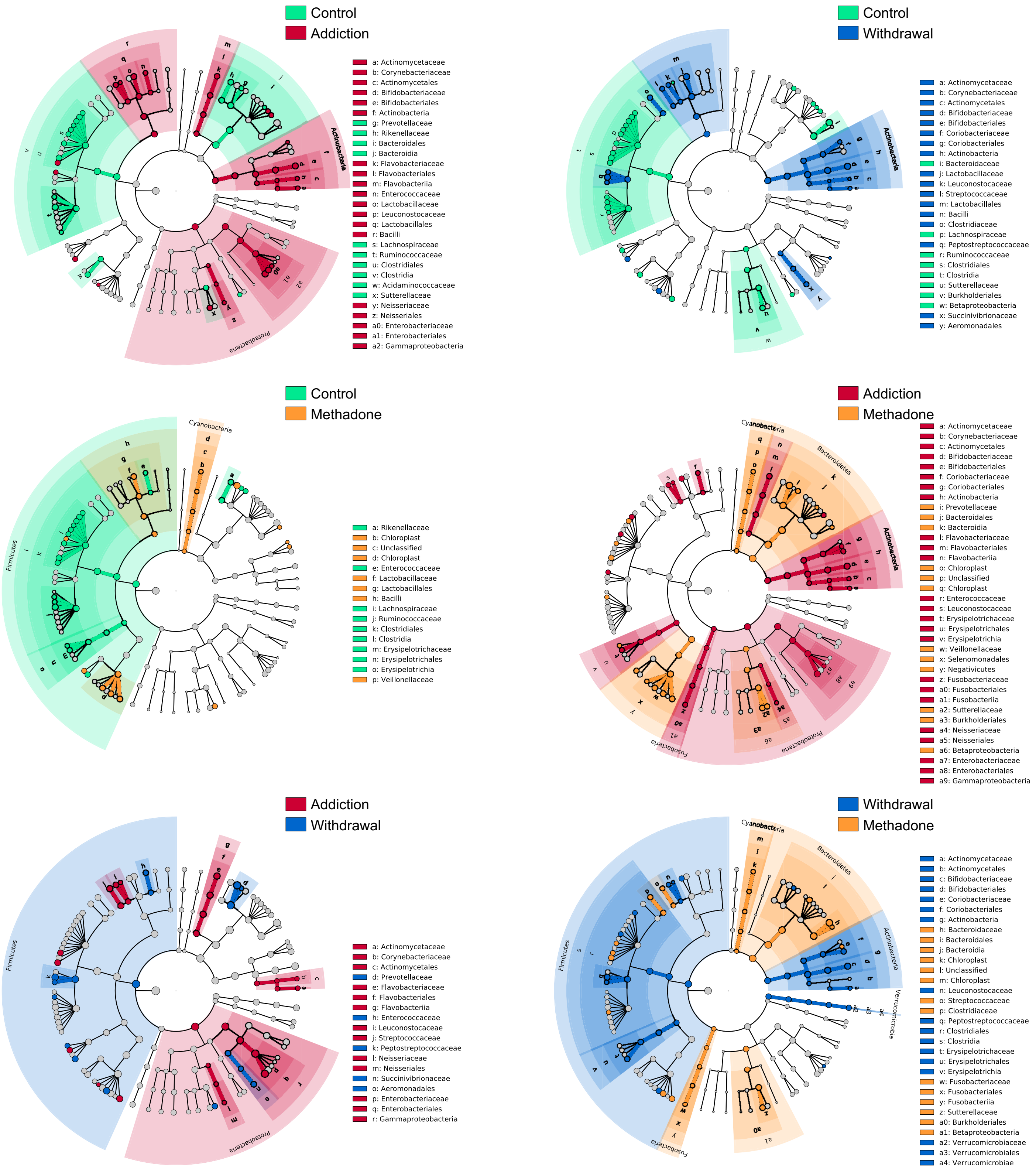

Figure S3. The results of linear discriminant analysis (LDA). LDA effect size (LEfSe) cladogram and LDA scores showing differentially abundant taxonomic clades with LDA scores > 2.0 and $p < 0.05$ among pair-wise comparisons.

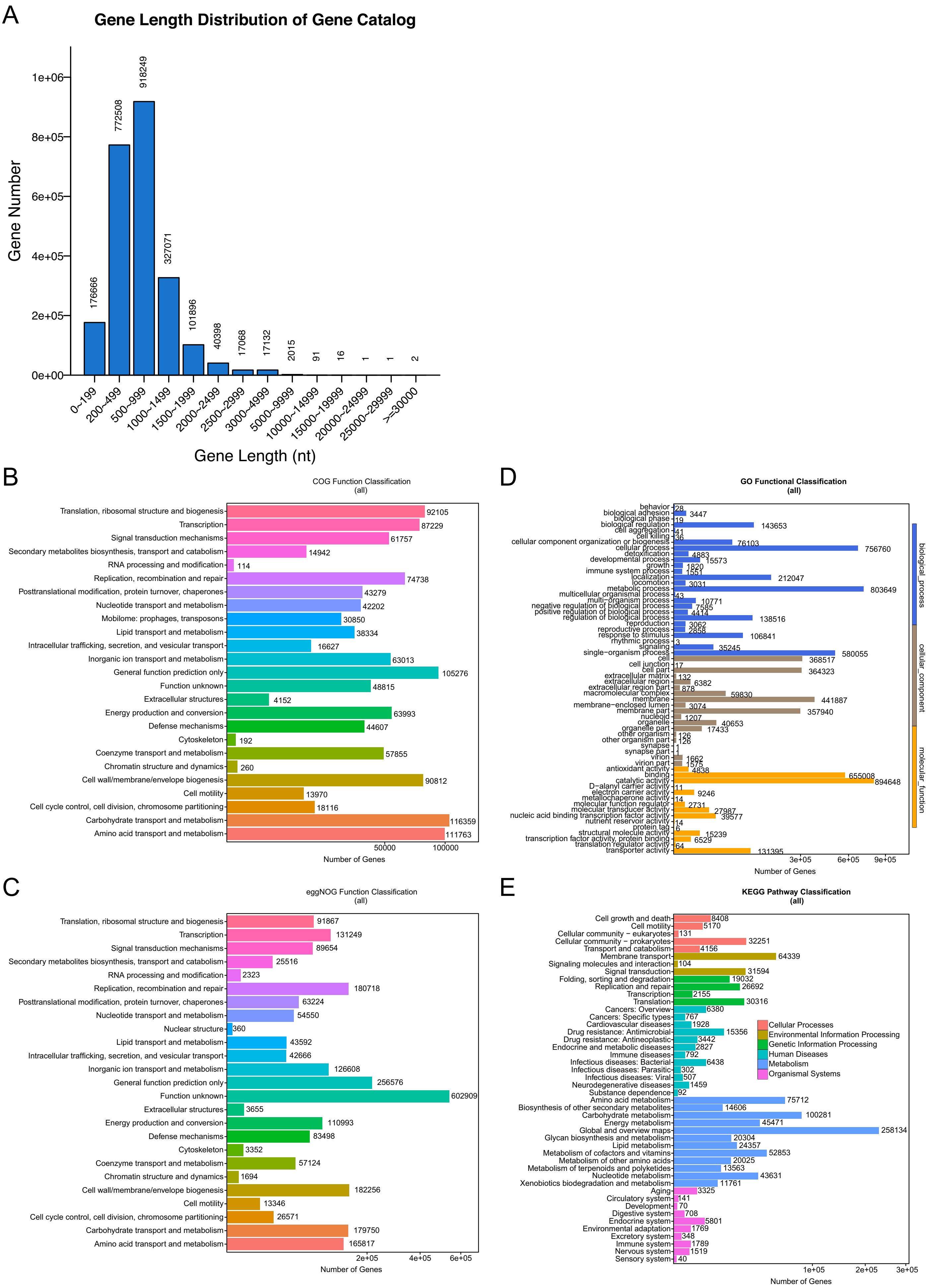

Figure S4. Summary of gene function annotation based on different databases. (A) Length distribution of genes. The X-axis indicates the length intervals and the Y-axis represents the number of genes. (B) COG

functional classification. Annotated genes were classified into several functional categories according to their COG annotation. (C) Summary of eggNOG database annotation. The orthologous groups are annotated with functional description lines with functional categories. (D) GO functional classification. The numbers of annotated genes were calculated for each GO term under the three ontologies. (E) Summary of KEGG annotation. The X-axis represents the number of genes annotated from each pathway and the Y-axis displays annotated pathways in particular subclasses.

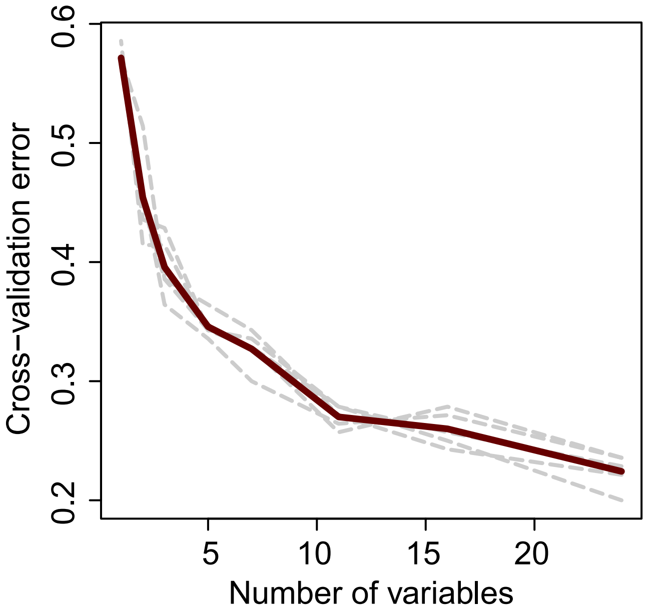

Figure S5. Distribution of five trials of 10-fold cross-validation error of the 24 featured bacteria genera in the random forest model. The gray and crimson curves indicate the five trials and the average cross-validation errors, respectively.

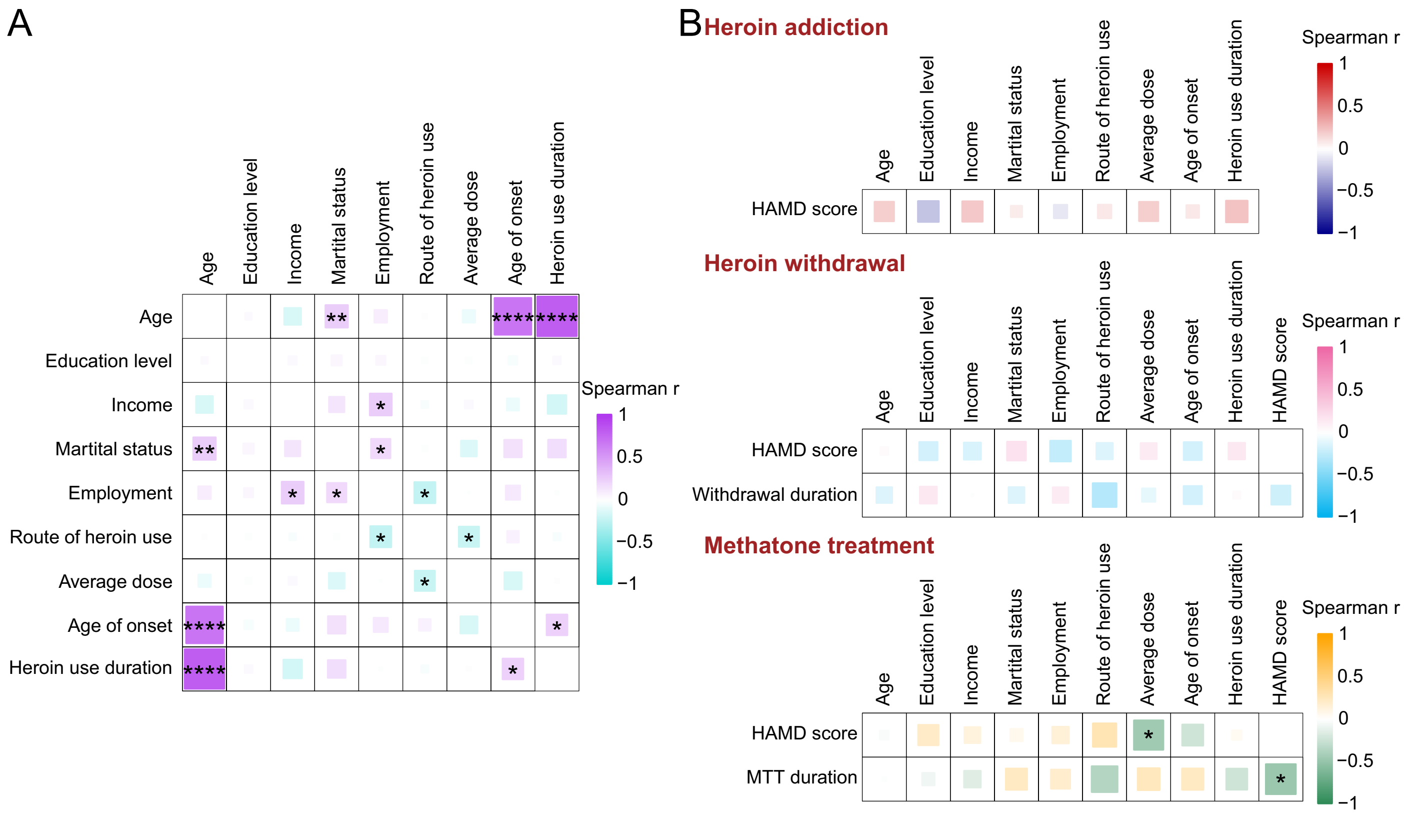

Figure S6. Correlation analysis for demographic and heroin abuse characteristics in HUD patients. (A) Spearman's bivariate correlation matrices for demographic and heroin abuse characteristics of HUD patients. Purple and cyan for positive and negative correlation, respectively. * $p < 0.05$, ** $p < 0.01$ and **** $p < 0.0001$. (B) Heatmap of Spearman's bivariate correlation coefficient among the basic information, HAMD score and withdrawal/MMT duration in different states of HUD patients. Red, pink and orange indicate mean positive correlation, and darkblue, skyblue and green mean negative correlation. * $p < 0.05$.
